# Experimental study on the punching failure model of soft red rock subgrade by BPT-BVM methods and it’s assessment of the load bearing capacity

**DOI:** 10.1038/s41598-024-61838-1

**Published:** 2024-05-14

**Authors:** Jibin Chen, Qiang Pan, Yao Wei, Yibin Luo, Zhuangfu Zhao, Li Zhao, Yu Bai

**Affiliations:** 1 Department of Civil Engineering, Chengdu Technological University (Yibin Campus), Yibin, 644000 China; 2 Chengdu Technological University, Chengdu, 611730 China; 3 Zhongyan Technology Co., Ltd, Beijing, 101100 China; 4 Beijing University of Technology, Beijing, 101124 China; 5 China Southwest Geotechnical Investigation and Design Institute CO.LTD, Chengdu, 610052 China; 6 School of Mechatronic Engineering, Southwest Petroleum University, Chengdu, 610500 China

**Keywords:** Punching failure, Moderate weathered mudstone, BPT, BVM system, Load bearing capacity, Civil engineering, Natural hazards, Mechanical properties

## Abstract

The recommended bearing capacity of medium weathering mudstone foundation is less than the capacity of the rock structure to withstand loads in Southwest China. A comprehensive failure characterization of medium weathering mudstone in Chengdu has been performed including bearing plate test (BPT), binocular vision measurement (BVM) test, uniaxial compressive strength test, trial trench test of shallow rock surface and 3D imaging in this paper. Failure behavior of rock has been modeled with 3D imaging algorithm that utilizes Zhang’s calibration method in BVM system combination with trial trench test of shallow rock surface. The bearing capacity of medium weathering mudstone foundation were extracted from uniaxial experiments and BPT-BVM test by fitting relevant material properties to the data. The results revealed that: Bearing capacity of medium weathering mudstone of layered isotropic in Chengdu is undervalued. Specifically, the characteristic load carrying value is in the range 1500–2500 kP, that is 50% higher than in the local standard system. Failure process is different from Hoek–Brown Failure Criterion, presenting a wave peak transfer phenomenon of the increment displacement into the distance. Thus, it can be reduced to that of punching failures for thin bedded structures of Moudstone foundations. Compressive strength of soft rock proves to be main factor limiting the bearing capacity, a clear correlation between the uniaxial compressive strength reduction coefficient and the bearing capacity has been used to establish, leading to the proposal of a load bearing capacity prediction model.

## Introduction

Moderate weathered mudstone is a type of typical red soft rock mass that are widely distributed across the basin of Sichuan Province and its marginal areas in China^1^. The integration of Chengdu metropolitan area and Chengdu-Chongqing economic circle inevitably leads to the use of soft rock foundations in the construction of high and super-rise buildings in the region. The question of how to assess the bearing capacity of this type mass is of great engineering importance for the selection and optimization of building foundations.

The load bearing capacity of the soft rock foundation is mainly obtained by the regional engineering experience^2–4^ and the method of reducing the uniaxial compressive strength^4,5^. Engineering practices reveal that the load bearing capacity, as per regional engineering knowledge, falls short of the rock mass structure’s capacity, it indicating that the foundation’s design exhibits significant redundancy^6–8^. Academics are also aware of the implications of such problems for engineering, and proceeds to deduce geotechnical load bearing theory from the rock failure model. Song^9^ divides the failure of rock masses into three main types on the basis of controlling theory of rock structure, there are shear failure or punching failure for two-layer foundations and shear failure for isotropic rock foundation. Failure modes of single-multilayered rock masses, with each layer characterized by different material properties, are complicated. Liu et al.^10^ found that the peak stress of sandstone can be predetermined based on the crack initiation stress or the crack damage stress. Jing et al.^11^ identified the micro mesoscopic creep damage evolution and failure mechanism of sand mudstones. Zhao et al.^12^ pointed out that there is thrust rupture, thrust shear failure, and Prandtl failure in the lower void foundation. Bai et al.^13^ described the progressive failure of the moderately weathered mudstone into compaction, elasticity, elasto-plasticity, plasticity, and failure. However, the failure mode of the soft-rock foundation is linked to the compaction, weathering, temperature and micromacro-physical mechanics of the rock mass^12,14–26^. The failure mode of the homogeneous or anisotropy rock foundation may not be appropriate for layered isotropic soft rock mass in Southwest, China. There are still differences in the failure mode of soft rock foundations at present, and theoretical research remains stagnant. Therefore, it is suggested that the reduction method based on uniaxial compressive strength should be used for a simple estimation in guidelines^1–4^. Unfortunately, the method does not agree well with the results of in-situ load tests of rock foundations. For example, the recommended the load bearing capacity of the moderate weathered mudstone (*f*_ak_) subgrade is consistently 800–1200 kPa^2^ in Chengdu, which is significantly lower than the value of the plate loading test (> 1500 kPa)^1,4,8^. The reason is that failure mechanism of mudstone foundation cannot really be reflected by compressive strength testing, which results in the poor bearing capacity adequacy based on the reduction in rock compressive strength and poor agreement with load test results. The theory can not truly reflect the load-bearing failure mechanism of mudstone foundations, and practice lacks experimental demonstration based on load testing and direct rock strain testing, which severely underestimates the load-bearing capacity of soft rock^1,4,13,21,27^. In practice, Zeng et al.^28^, Peng^29^, Liu et al.^30^ have been gradually modifying the standards based on the load test of rock foundation, point load test, pressuremeter test, etc. However, due to regional restrictions, the above modifications cannot be widely distributed^4^. Furthermore, although method of bearing plate results are considered to be the most true and reliable, it is not widely popularized due to poor timeliness and the inadequacy of intelligent control^1,31,32^. At these points, it remains to discuss the appropriate computational method for soft red rock. Meanwhile, the unknown geometry model of the subsurface fractured rock mass and the lack of micro-mechanical mudstone parameters, the failure mechanism of the mudstone foundation cannot be truly reflected^33,34^. The failure pattern of the moderate weathered mudstone foundation in soft rock, especially layered isotropic soft rock in Chengdu, is still not clear. When a higher top load is present, the engineering cost of the foundation treatment method is significantly increased if the value is achieved according to the specification, which does not achieve the dual carbon and economic objectives.

On this basis, this paper takes the foundation project of STI (Science and Technology Innovation) Park in Tianfu New District of Chengdu as a case, the in-situ Bearing Plate Test (BPT) with Binocular Vision Measurement (BVM) is used to dynamically monitor the spatial range of rock mass deformation under vertical loading conditions of moderate weathered mudstone. Monitoring results combine shallow rock surface trough test and 3D imaging to analyze the failure mode of the mudstone foundation. Then, according to the difference between the in-situ loading method and the uniaxial compressive strength test of the same condition rock among 100 sites, this paper presents an empirical formula for calculating the load bearing capacity with both shear and compressive effects and suggests the value of the computational parameters. The results of this research are useful in updating the foundation design theory and provide valuable empirical evidence to give full play to the bearing capacity of the soft red stone.

### Characteristics of mudstone in the study area

**Figure 1:**
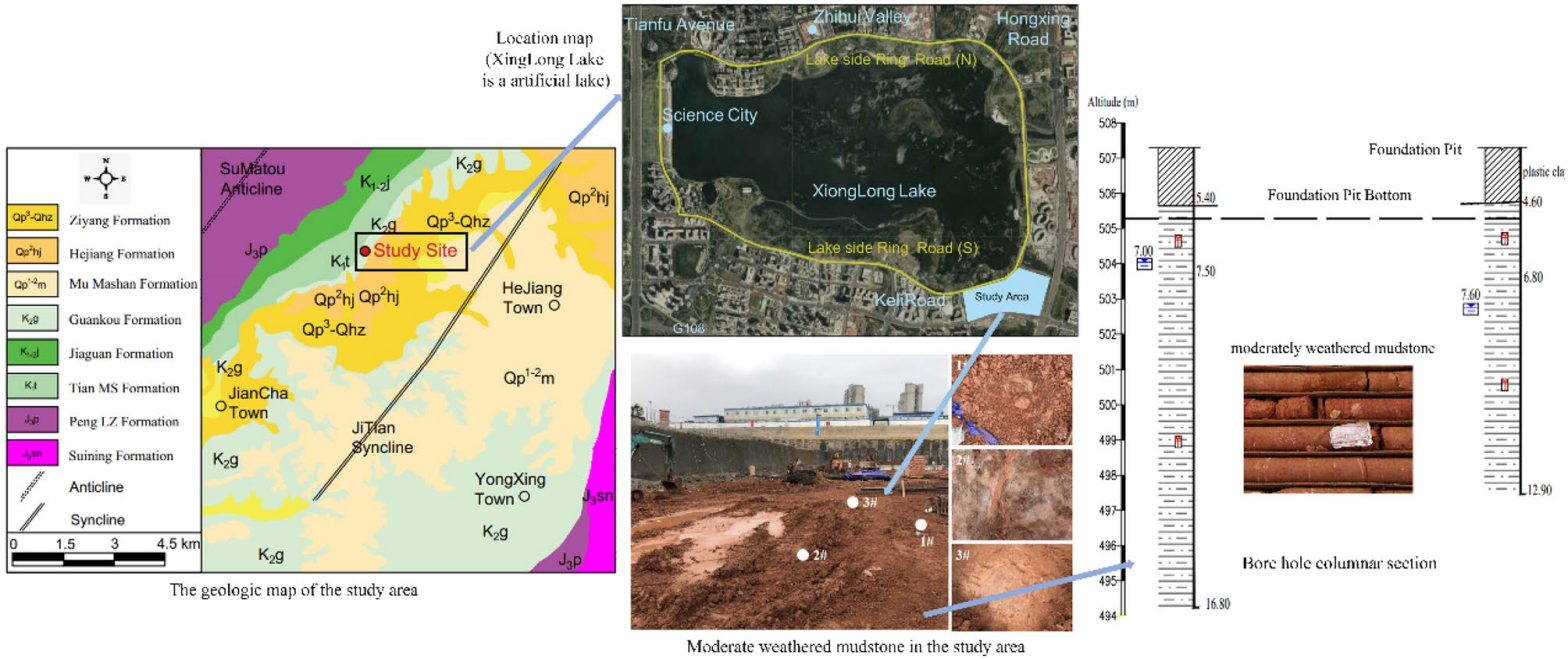
Study site conditions.

The research site is located at south of the Xinglong Lake in Tianfu New Area of Chengdu, China. The geomorphic units of the site belong to the third terrace of Chengdu Plain of Min River Basin, with elevations ranging from 494.21 to 507.02 m. The formation of the site is dominated by the moderately weathered mudstone of the Guankou Formation (K_2g_) in Upper Cretaceous (as seen in Fig. 1). The formation consists of a brownish-red rock with a thin layer 10 to 15 cm in thickness, the rock mass within a range of 10 m below the bottom of the foundation pit is moderately weathered mudstone (the BPT and BVM tests are conducted after the excavation of the foundation pit is completed). The density of the rock is 23.0 kN/m^3^, the natural compressive strength of the rock ranges from 2.67 to 2.79 MPa, the rock mass index (RQD) is 50 to 80, and the basic rock mass grade is V. Analysis of rock samples using X-Ray Diffusion and X-ray fluorescence revealed that the mudstone in the study area contained quartz, illite, aluminite and black mica. The major constituents of the rock sample elements were SiO_2_ (54.61 wt%), Al_2_O_3_ (19.54 wt%), CaO (7.16 wt%). In general, the lithology of the moderately weathered mudstone in the study area is soft, depending on Mohs’ hardness scale^35^. The moderately weathered mudstone is a layered isotropic rock mass.

**Figure 2:**
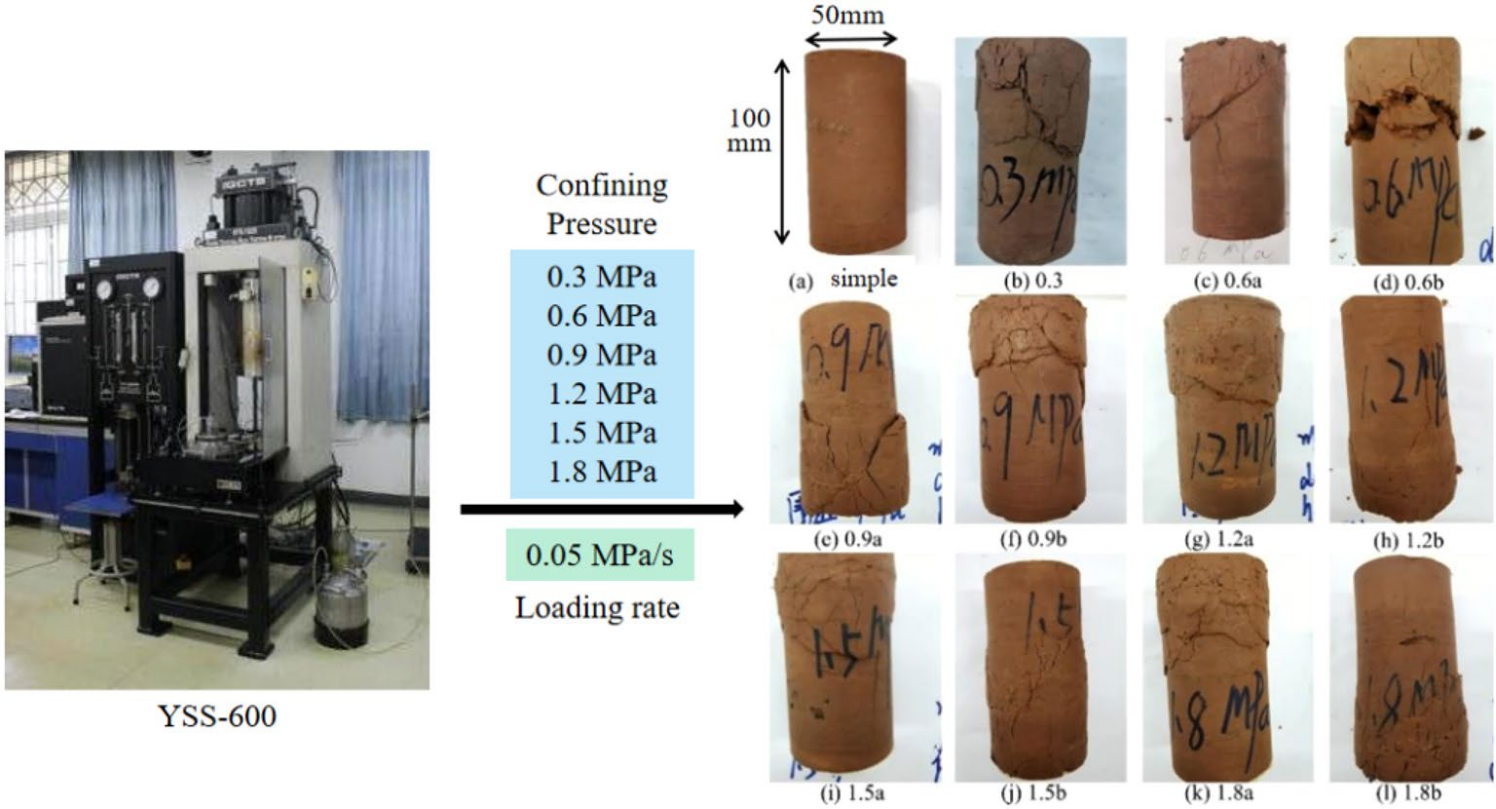
Triaxial compression test.

**Figure 3:**
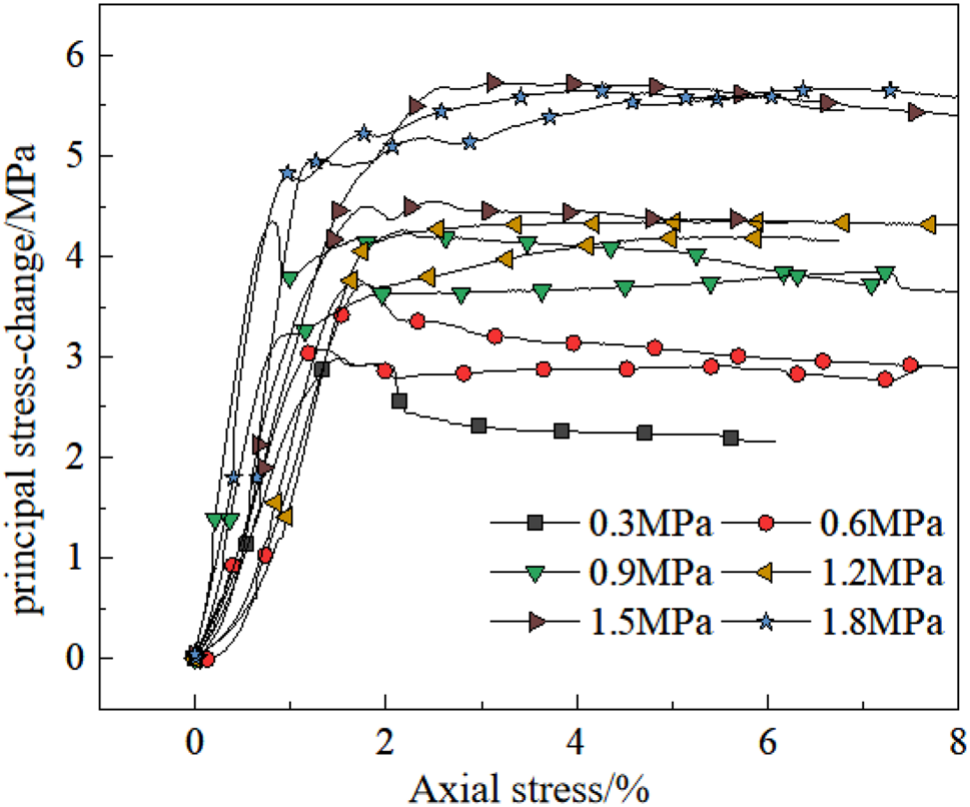
Stress–strain curves of triaxial compression.

**Figure 4:**
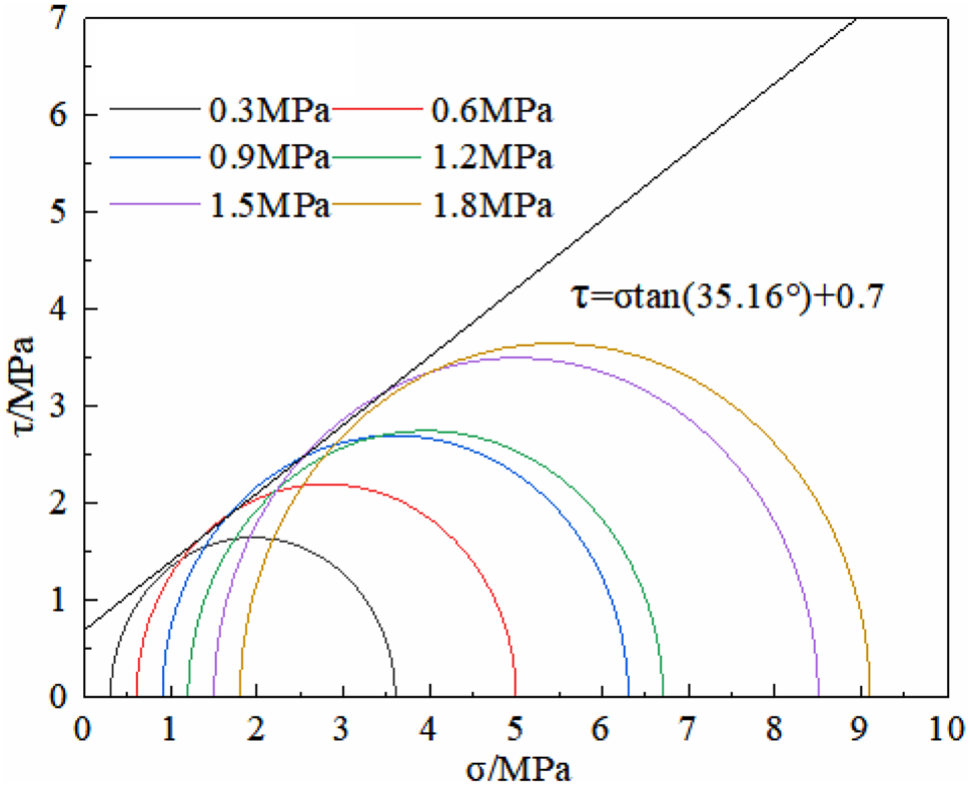
Mohr’s stress circle and strength envelope of mudst.

In order to determine the strength of triaxial rock compression, the YSS-600 triaxial rock mechanics test system was used to failure mudstone samples under different conditions of confining pressure (as seen in Fig. 2). 12 samples were got for indoor test, which were from the depths of 505.5 m and 503 m and the state of the rock in its natural state. Typically, samples measure 10 cm in height and 5 cm in diameter, with their dimensions detailed in Table 1. The confining pressure range of this research is preliminarily determined according to the actual excavation depth, and the upper load of the foundation. The strength of the sample in field was low^13^. So, the confining pressure is characterized by 6 gradients: 0.3 MPa, 0.6 MPa, 0.9 MPa, 1.2 MPa, 1.5 MPa and 1.8 MPa, respectively (parallel experiments a and b under pressure in each group were conducted)^13^. For the rock mass test, a loading rate of 0.05 MPa/s was applied to the machine, and synchronously apply confining pressure and axial pressure at pre-set values of the disturbance. A continuous loading method is used in which the axial load and axial strain are measured step-by-step until the specimen fails, and the failure load of each specimen is recorded. The stress–strain curves of the triaxial tests are shown in Fig. 3. The strength of the sample increased with the increase of confining pressure, and the peak strength increased from 3 to 5.5 MPa. When the confining pressure was low (0.3–0.6 MPa), the mudstone exhibited strain softening after it reached the peak of the main stress difference. There was no change in the main difference in stress after reaching the peak when the confining pressure was greater than 0.9 MPa. When the axial strain comes to 8%, there were many cracks in the upper part of the rock sample but no apparent cracks in the lower part of the rock sample (as seen in Fig. 2). The whole-rock sample was not crushed^13^. Combined with the stress molar circle and the resistance envelope of the mudstone at the site (Fig. 4), in the study area the angle of friction and cohesion in the rock mass were calculated to be 35.16° and 0.7 MPa, respectively.

**Table 1:** Rock sample dimensions.

No.	Hight/mm	Diameter/mm	Weight/g	Number of items
1-①	98.32	49.70	400.61	2
1-②	101.68	50.42	416.87	2
2-①	89.71	50.29	376.47	2
2-②	102.71	49.48	435.99	2
3-①	106.72	50.36	448.10	2
3-②	77.32	50.25	314.01	2

1: test site No. ①: Sampling depth 505.5 m; ②: Sampling depth 503 m.

### In-situ test plan

Three BPTs with the numbers 1 #, 2 #, and 3 # were performed at the site on the basis of the guideline “GB/T50266 ”^2^ and “GB50007”^3^ (as seen in Fig. 1). Each test site was spaced between 30 and 50 m apart, and the rock characteristics of the test site varied slightly which was related to the light conditions, ambient humidity conditions and excavation exposure at the different test phases. The BVM system is fed into Bearing Plate Tests at the site. This method can monitor surface deformation of rock mass in real-time, track the rock mass progressive failure degree and image rock mass in 3D deformation failure mode.

### BVM system

A displacement measurement system based on BVM consists of both hardware and software. Each hardware component consists of a binocular camera, a data storage, processing computer, and a customized target. A 4 mm to 12 mm zoom lens is attached to the binocular camera, with a maximum resolution of 2560 × 960 pixels, and a tunable acquisition frame rate of up to 60 frames per second. It is possible to adjust the stereo baseline from 4.5 to 18 cm, and the camera field of view ranges from 29° to 78°. The vision system was worked on a laptop. The movements of a target can be recorded and tracked by the camera and synchronously transferred to the computer, where the displacement is calculated using object centre location algorithms and coordinate transformations^36–40^. The process of BVM include scene layout, calibration, stereo matching, and a three-dimensional coordinate solution.

**Figure 5:**
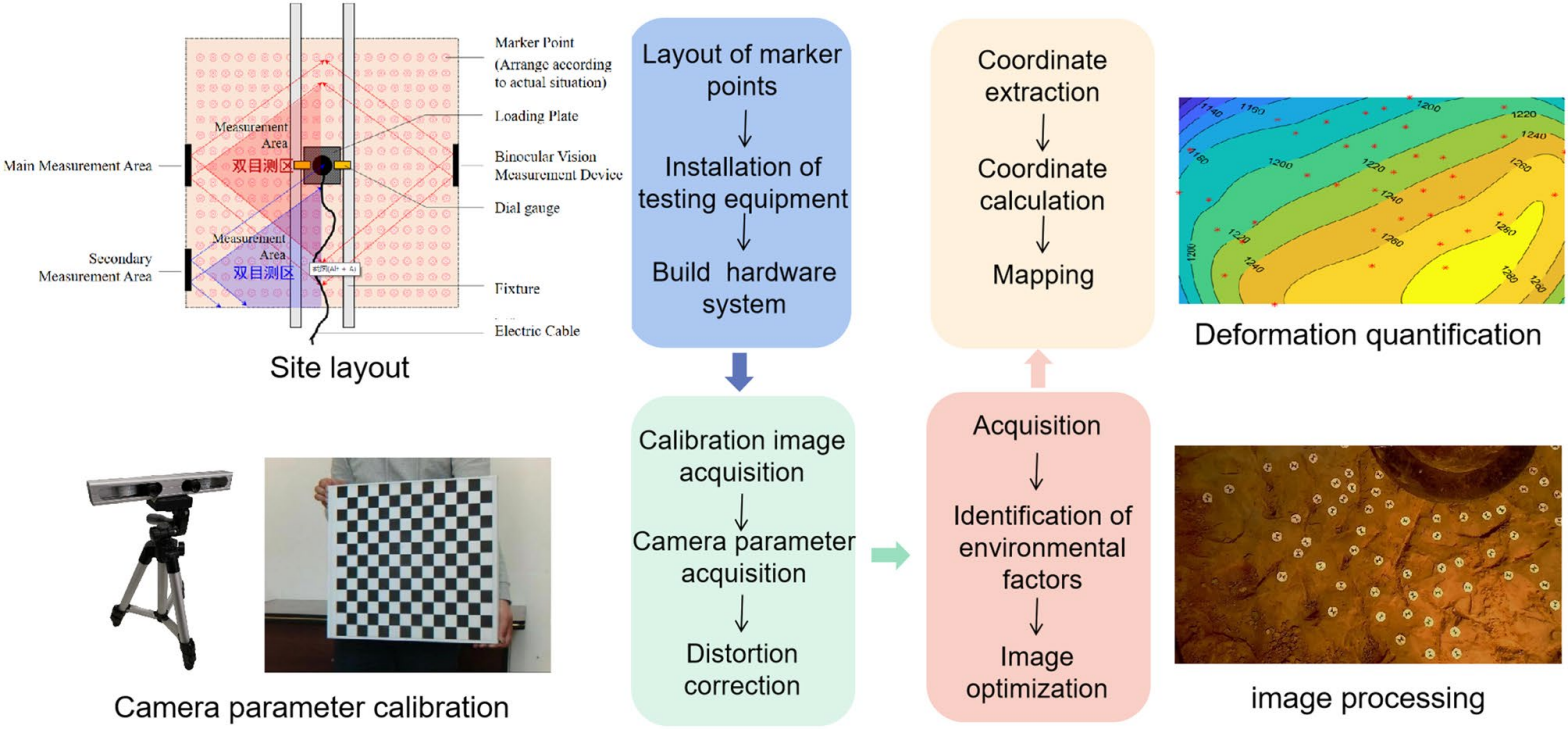
Process of binocular vision measuring.

In the site layout phase, the site is divided into four measurement areas, each of which radiates a number of points of interest. A schematic of the layout of the BVM system is shown in Fig. 5.

**Figure 6:**
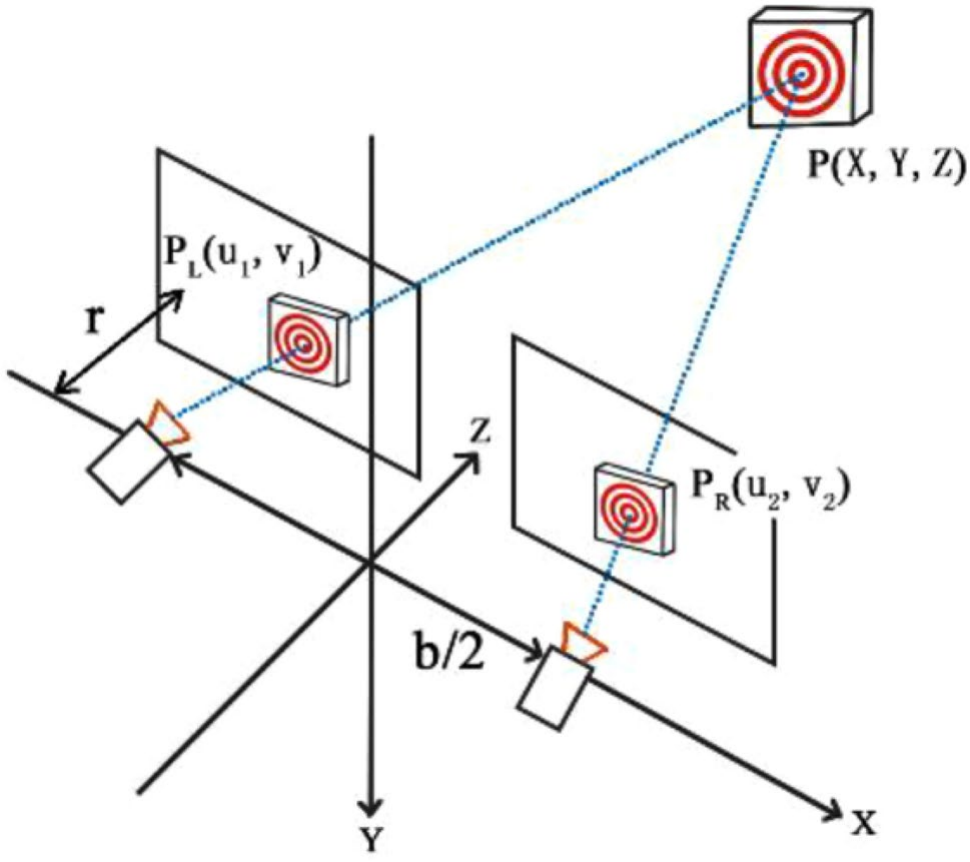
Zhang’s calibration method.

Camera calibration during the tracking process uses Zhang’s calibration method^36,41^, as seen in Fig. 6. Equation (1) was used to extract the 2D coordinates of the center of the butterfly shaped markers^41^.

1 $$ R(x,y) = ax^{2} + by^{2} + cxy + dx + ey + f $$

where R (x, y) is the response function of the angular point, which detects nine pixels around the angular point, thus creating a set of six unknowns (a, b, c, d, e, f) that are super determinate and then solved for using the least squares method. The coordinates of the point (x, y) are found by deriving a quadratic polynomial using Eq. (2).

2 $$ \left\{ {\begin{array}{*{20}c} {\frac{\partial R}{{\partial x}} = 2ax + cy + d = 0} \\ {\frac{\partial R}{{\partial y}} = 2by + cx + e = 0} \\ \end{array} } \right. $$

Zhang’s calibration method^41^ states that two coefficient matrices can be constructed by calibration of a binocular camera. The coordinates of the left and right image pixels are then combined using a coefficient matrix to solve the over-determined equations and obtain the spatial coordinates. After obtaining the spatial coordinates of the target in each frame of the image, the value of the displacement of the measurement point can be quantified to give the surface deformation of the talus. Figure 3 shows the computational principle. Formulas (3) and (4) are available in terms of the ideal linear camera model^37^.

3 $$ {\mathbf{Z}}_{{{\mathbf{C1}}}} \left[ {\begin{array}{*{20}c} {{\mathbf{u}}_{{\mathbf{1}}} } \\ {{\mathbf{v}}_{{\mathbf{1}}} } \\ {\mathbf{1}} \\ \end{array} } \right] = {\mathbf{P}}_{{\mathbf{1}}} \left[ {\begin{array}{*{20}c} {\mathbf{X}} \\ {\mathbf{Y}} \\ {\mathbf{Z}} \\ {\mathbf{1}} \\ \end{array} } \right] = \left[ {\begin{array}{*{20}c} {{\mathbf{p}}_{{{\mathbf{00}}}}^{{\mathbf{1}}} } & {{\mathbf{p}}_{{{\mathbf{01}}}}^{{\mathbf{1}}} } & {{\mathbf{p}}_{{{\mathbf{02}}}}^{{\mathbf{1}}} } & {{\mathbf{p}}_{{{\mathbf{03}}}}^{{\mathbf{1}}} } \\ {{\mathbf{p}}_{{{\mathbf{10}}}}^{{\mathbf{1}}} } & {{\mathbf{p}}_{{{\mathbf{11}}}}^{{\mathbf{1}}} } & {{\mathbf{p}}_{{{\mathbf{12}}}}^{{\mathbf{1}}} } & {{\mathbf{p}}_{{{\mathbf{13}}}}^{{\mathbf{1}}} } \\ {{\mathbf{p}}_{{{\mathbf{20}}}}^{{\mathbf{1}}} } & {{\mathbf{p}}_{{{\mathbf{21}}}}^{{\mathbf{1}}} } & {{\mathbf{p}}_{{{\mathbf{22}}}}^{{\mathbf{1}}} } & {{\mathbf{p}}_{{{\mathbf{23}}}}^{{\mathbf{1}}} } \\ \end{array} } \right]\left[ {\begin{array}{*{20}c} {\mathbf{X}} \\ {\mathbf{Y}} \\ {\mathbf{Z}} \\ {\mathbf{1}} \\ \end{array} } \right] $$

4 $$ {\mathbf{Z}}_{{{\mathbf{C2}}}} \left[ {\begin{array}{*{20}c} {{\mathbf{u}}_{{\mathbf{2}}} } \\ {{\mathbf{v}}_{{\mathbf{2}}} } \\ {\mathbf{1}} \\ \end{array} } \right] = {\mathbf{P}}_{{\mathbf{2}}} \left[ {\begin{array}{*{20}c} {\mathbf{X}} \\ {\mathbf{Y}} \\ {\mathbf{Z}} \\ {\mathbf{1}} \\ \end{array} } \right] = \left[ {\begin{array}{*{20}c} {{\mathbf{p}}_{{{\mathbf{00}}}}^{{\mathbf{2}}} } & {{\mathbf{p}}_{{{\mathbf{01}}}}^{{\mathbf{2}}} } & {{\mathbf{p}}_{{{\mathbf{02}}}}^{{\mathbf{2}}} } & {{\mathbf{p}}_{{{\mathbf{03}}}}^{{\mathbf{2}}} } \\ {{\mathbf{p}}_{{{\mathbf{10}}}}^{{\mathbf{2}}} } & {{\mathbf{p}}_{{{\mathbf{11}}}}^{{\mathbf{2}}} } & {{\mathbf{p}}_{{{\mathbf{12}}}}^{{\mathbf{2}}} } & {{\mathbf{p}}_{{{\mathbf{13}}}}^{{\mathbf{2}}} } \\ {{\mathbf{p}}_{{{\mathbf{20}}}}^{{\mathbf{2}}} } & {{\mathbf{p}}_{{{\mathbf{21}}}}^{{\mathbf{2}}} } & {{\mathbf{p}}_{{{\mathbf{22}}}}^{{\mathbf{2}}} } & {{\mathbf{p}}_{{{\mathbf{23}}}}^{{\mathbf{2}}} } \\ \end{array} } \right]\left[ {\begin{array}{*{20}c} {\mathbf{X}} \\ {\mathbf{Y}} \\ {\mathbf{Z}} \\ {\mathbf{1}} \\ \end{array} } \right] $$

(u1, v1, 1) and (u2, v2, 1) are the pixel homogenization coordinates of the points P1 and P2 in the images on the left and right side, respectively. (X, Y, Z, 1) is the homogeneous coordinate of P in the global coordinate system. *P*1 ii and *P*2 ii are matrices of coefficients, which can be obtained from the matrix of the internal and external camera parameters. During load testing, the least square method is used to find the surface deformation of the rock mass about the bearing plate through the solution of Eq. (5).

5 $$ \begin{gathered} \left( {u_{1} p_{20}^{1} - p_{00}^{1} } \right)X + \left( {u_{1} p_{21}^{1} - p_{01}^{1} } \right)Y + \left( {u_{1} p_{22}^{1} - p_{02}^{1} } \right)Z = p_{03}^{1} - u_{1} p_{23}^{1} \hfill \\ \left( {v_{1} p_{20}^{1} - p_{10}^{1} } \right)X + \left( {v_{1} p_{21}^{1} - p_{11}^{1} } \right)Y + \left( {v_{1} p_{22}^{1} - p_{12}^{1} } \right)Z = p_{13}^{1} - v_{1} p_{23}^{1} \hfill \\ \left( {u_{2} p_{20}^{2} - p_{00}^{2} } \right)X + \left( {u_{2} p_{21}^{2} - p_{01}^{2} } \right)Y + \left( {u_{2} p_{22}^{2} - p_{02}^{2} } \right)Z = p_{03}^{2} - u_{2} p_{23}^{2} \hfill \\ \left( {v_{2} p_{20}^{2} - p_{10}^{2} } \right)X + \left( {v_{2} p_{21}^{2} - p_{11}^{2} } \right)Y + \left( {v_{2} p_{22}^{2} - p_{12}^{2} } \right)Z = p_{13}^{2} - v_{2} p_{23}^{2} \hfill \\ \end{gathered} $$

### BPT with BVM method program

**Figure 7:**
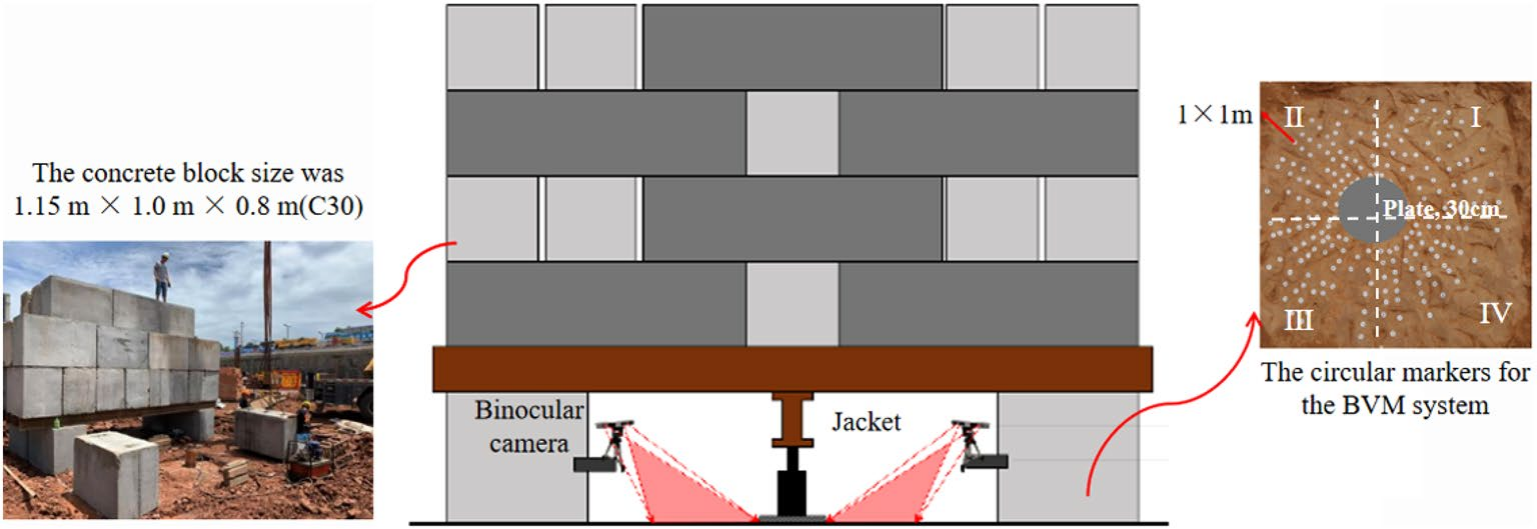
Test set-up.

Prior to testing, each of the three locations was manually smoothed to a range of 1 m × 1 m and used to position the bearing plates (plate diameter is 300 mm) as well as to attach the circular markers for the BVM system to be fabricated (Fig. 7). The bearing plates were placed in the center of the polished area and then partitioned according to four quadrants of the coordinate system at rectangular coordinates, with 40–49 marker points adhered to each of the surfaces. A layer of fine sand is laid between the plate and the ground, with a thickness of about 1 cm. It can minimize the influence of the interface between plate and ground. At the top, the overloading tonnage is 120 T (approximately 17,000 kPa) and is loaded in steps of 300 kPa per stage. Figure 7 shows a representative photograph of the testing process at each point.

Because the BVM results must be processed in the laboratory, four micrometres are installed around the holder simultaneously during the test in order to ensure the control ability of the experimental data as well as to monitor the displacement according to the Standards^2,42^. Basic data were read once immediately after loading and then every 10 min thereafter; if the difference between 3 consecutive readings does not exceed 0.01 mm, the next charge is loaded. Only if the foundation is damaged or the increment in displacement of two loads is more than five times, the test can be terminated, then unloaded in a stepwise manner. The timing of the data recording in the BVM system is based on the timing of the above readings. Data processing and analysis were performed at the end of the experiment in three parts. (1) From the p-s curve, the prior grade at the end of the linear phase (or elastic stage) of the curve is taken to be the proportional limit load (Pa). The forward load at the end of the test is taken as the ultimate load (Pu). Then the load bearing capacity of the moderate weathered mudstone can be seen as *f*_*ak*_ = min{P_a_, P_u_/3}; (2) Revealing features of destruction of deep bedrock foundations. To reveal the overall level of failure at the test site, shallow surface troughs were manually excavated to obtain mass damage from the deep rock; (3) Coordinate solution and spatial imaging of the bedrock failure. At any given loading step, the surface displacement of the rock mass is characterized by the absolute value of the displacement increment, and rock mass deformation and displacement at any time are obtained.

### Test results

#### The results of BPTs

**Figure 8:**
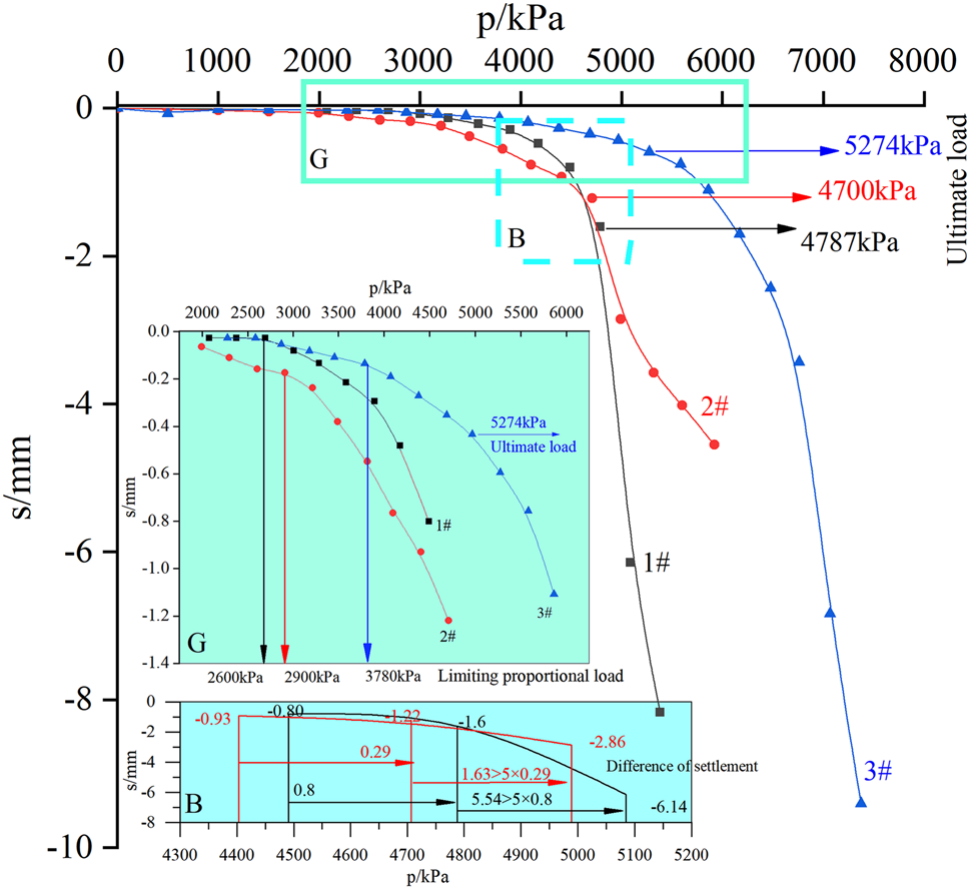
p-s curve.

The base of the p (Loading)—s (Settlement) curve on the load test results from three test points is shown in Fig. 8. As can be seen from Fig. 8, the vertical deformation of the rock mass is proportional to the vertical load. The p-s curve has three stages of deformation as the load increasing: the elastic stage, the elastoplastic stage and the failure stage. The first part of the p-s curve is linear and slowly varying, indicating that the stiffness of the rock mass is high and capable of withstanding significant loading; Once the load reaches a certain value, the area of plastic deformation becomes progressively wider, and the p-s curve begins to curve in a linear fashion, indicating that the stiffness of the rock decreases and the local hardness increases; The p-s curve clearly shows a turning point as the foundation loses its load bearing capacity. So, the load bearing capacity characteristics of the rock mass may be driven by the changing trend in the curve.

At test point 1#, no significant inflection point was observed in the curve prior to the loading of 4000 kPa. As the load is increased beyond 4000 kPa, the displacement of the rock mass foundation increases and the curve shifts from elastic to elastoplastic. Relative to the load 4780 kPa, when the load is raised to 5000 kPa, the foundation has a displacement increment of about 5 times and a total displacement of 6.1 mm. According to GB50007^3^ guideline, the limiting proportional load is about 2600 kPa, the ultimate load is about 4780 kPa. Based on the results of this study, the characteristic value of the bearing capacity is min{2600 kPa, 4787/3 kPa} = 1595 kPa. The evolution of the curve profiles at the 2# test point is the same, the characteristic value of the bearing capacity is 1567 kPa. For 3#, the ultimate bearing capacity is determined by the second point of inflection of the curve, i. e., 1758 kPa.

Although the results vary slightly from one trial to another, the difference is less than 5%. The moderate-weathering mudstone bedrock on the site has a load-bearing capacity of 1640 kPa.

#### The results of shallow surface troughs and 3-D imaging

**Figure 9:**
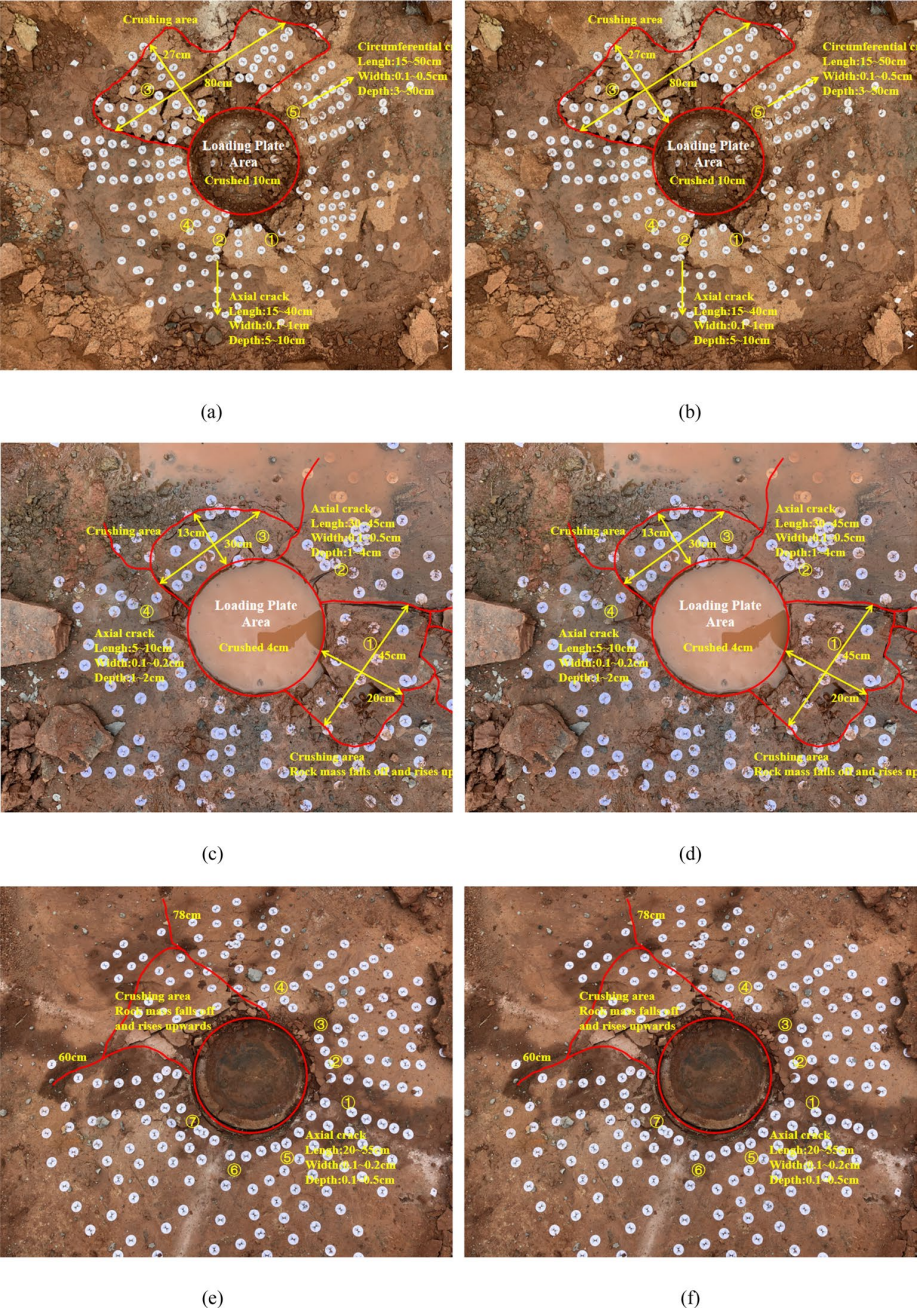
Failure characteristics of rock mass under vertical load. (**a**) Shallow failure of 1#, (**b**) Shallow cut of 1#, (**c**) Shallow failure of 2#, (**d**) Shallow cut of 2#, (**e**) Shallow failure of 3# and (**f**) Shallow cut of 3#

**Figure 10:**
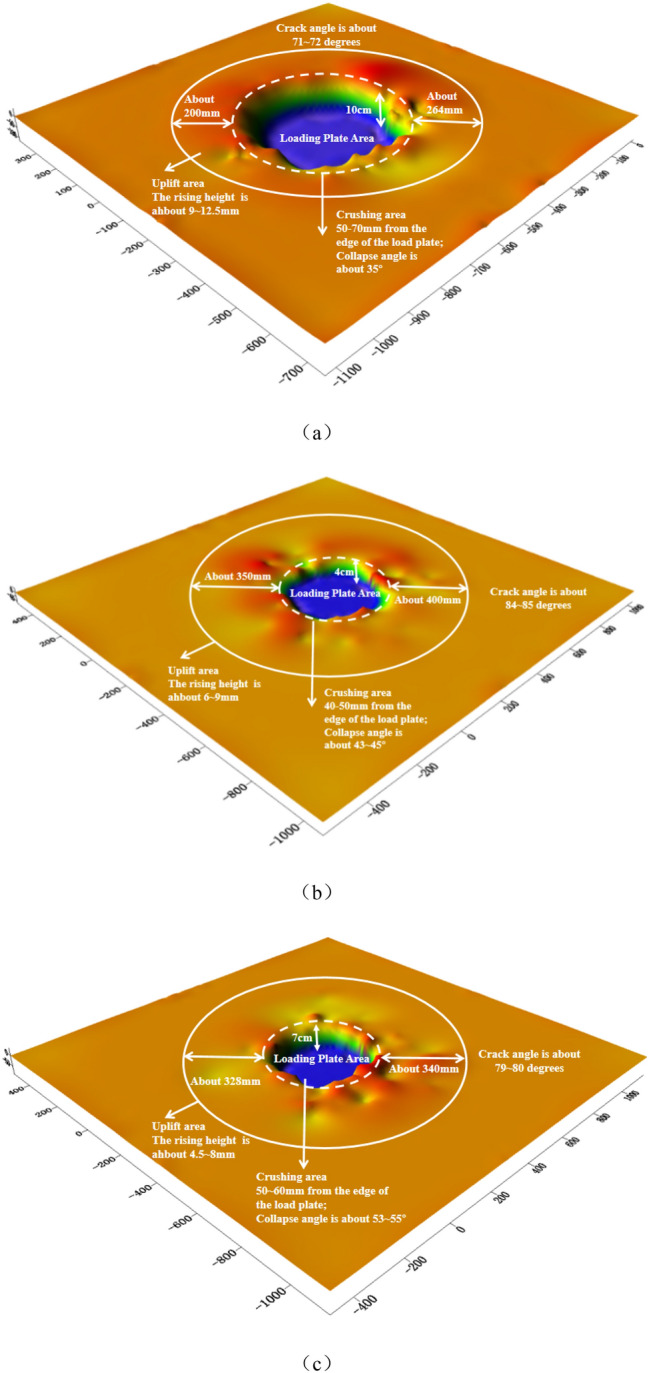
Three-dimensional imaging. (**a**) 1#, (**b**) 2# and (**c**) 3#

At the test site, the failure of the rock foundation is obtained by combining the surface deformation of the artificial rock being excavated (Fig. 9) with the 3D imaging of rock mass disrupted horizon (Fig. 10). From the Figures, failure state at all three test locations is very similar, including uplifted area, collapse settlement area and failure plane angle of inclination (the angle between the horizontal datum plane and the active wedge). Specifically for each test location:

At point 1 #, the outer-plate fracture development pattern (Fig. 9a,b) is composed of two major fractures with multiple secondary fractures present. The principal fractures are two nearly vertical, penetrating cracks in North/South and East/West, and the crack length varies between 20 and 40 cm. Secondary dendrite fractures derived from the main cleft are 10 to 20 cm long without significant regularity. It is important to note that the region in Fig. 9a,b severe damage to both sides of the cutting is attributed to pull jacks. After deep stripping, it is found that the rock mass directly below the bearing plate is fractured as a crushing failure, and the depth of failure is about 7 to 10 cm below the surface of the experimental site. This depth of subsidence is broadly consistent with the monitoring findings. The entire failure zone has a range of influence of about 1.2 m diameter, and the depth of fracture downward is approximately the same as that for compression plate crushing at the center of the circle.

At point 2 #, the pattern of development of outer-plate fracture (Fig. 9b,c) is similar to that observed at point 1#. The fissure on the south side is about 30 cm long and 3 to 5 mm wide. The crack is approximately 45 cm long on the west side and 0.5 to 1 cm wide. In other regions, although large cracks are not obvious, small cracks are common, and there is no remarkable regularity. The rock mass directly beneath the bearing plate is found to be fractured as a crushing failure after deep stripping, and the depth of rupture is approximately 4–6 cm below the surface of the experimental site. In general, the failure zone is approximately 1 m in diameter and the extended depth of the cracks on either side is also approximately 5 cm below the surface.

At point 3 # (Fig. 9e,f), the development pattern of the outer-plate fracture is centered on the pressurized plate and scattered. The angle between the two adjacent cracks is approximately 45°, and the length of the fracture ranges from as much as 20 to 78 cm. After stripping, it was found that the rock mass below the load plate was fractured as a whole. The fracture depth was about 10 cm below the surface of the test site and the extension depth of the cracks was about 10 cm below the surface. The failure zone was defined as an ellipse (70 cm in diameter of long axis and 50 cm in diameter of short axis). At the same time, the mass of rock at Point 3# is found to be more intact than at the other test points, which explains the reason for the higher load-bearing capacity of it.

The deformation and failure characteristics of the shallow bedrock surface are as follows: The damage domain has an ellipse with a diameter of approximately 1 m, and the fracture pattern surrounding the plate consists of many vertical primary fractures with minor secondary fractures. The crack is about 20–78 cm long and about 3–5 mm wide. The deep exposed area is a compacted mass of rock as a whole, and the depth of extension of the fissures on each side is approximately equal to the thickness of the thin rock mass. As the plate deforms under load, the rock mass surrounding the plate experiences a vertical shear.

Combined with 3D imaging of rock mass failure (Fig. 10), the failure zone of the vertical bearing rock mass is ultimately determined. The relevant statistics are presented in Table 2. As can be seen in Fig. 10 and Table 2, (1) beneath the load plate, a dense rock mass has settled, reaching a depth approximately the thickness of the rock stratum; (2) the deformation of the rock mass around the outer plate is typically between 230 and 310 mm, with a maximum range of up to 450 mm, which is related to the length of the outer crack of the plate; (3) the uplift region is about 100 mm away from the edge of the plate, and the uplift altitude is 5 to 10 mm; (4) the angle between the horizontal datum plane and the active wedge is about 70° (up to a maximum of 80–85°), calculated by the average internal friction angle of the rock mass as 31.34°. In agreement with Hoek–Brown Failure Criterion^9,43,44^, the relationship between the actual failure zone and theoretical rupture depth by the failure plane angle of inclination is found to be irregular.

**Table 2:** A summary of bearing plate test results and the failure index.

No.	*f*_rk_/kPa	*f*_ak_/kPa	Failure zone		
			H_1_/cm	Lc/cm	D_1_/cm
1#	2730	1595	10	20–40	40–55
2#	2670	1567	4	30–45	30–45
3#	2790	1758	7	20–78	50–78

*f*_*rk*_: uniaxial compressive strength; *f*_*ak*_: characteristic value of bearing capacity; H_f_: failure depth; R_f_: diameter of failure zone; Lc: crack length.

### Displacement increment and cumulative displacement

**Figure 11:**
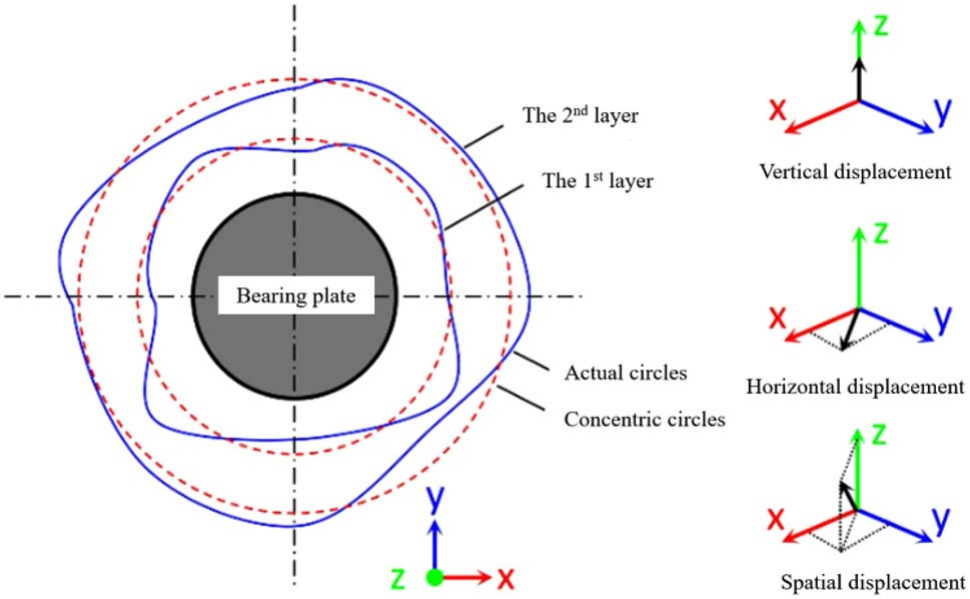
Diagram of circle arrangement^13^.

To reveal the failure characteristics of the medium-weathered mudstone foundations, a dynamic process calculation of the rock foundation surface deformation during loading is performed. Driven by the true smoothness of the rock mass, the average strain of different positions on the rock foundation surface is calculated by concentric circles (As seen in Fig. 11). Depending on the monitoring range, the rock mass deformation area is divided into six layers, and the spacing of each layer is approximately 20 to 30 mm. The displacement increment of each ring layer is defined as the average displacement of all markers in the same ring layer and the cumulative displacement increment is the cumulative displacement increment of the same ring layer with loading time^13^. The first to sixth layers gradually moved away from the bearing plate. Then, the 3D coordinates of the marking points in each layer of the test point at each time through the vertical, horizontal, and spatial displacement increment (the spatial deformation displacement of any point) of the area around the bearing plate were obtained. The vertical displacement increment was the absolute value of the Z-axis coordinate deformation. The X-axis and Y-axis indicate the deformation increment in the horizontal direction. The spatial distance between the X, Y, and Z axes was the spatial displacement increment. Significantly, the cumulative displacement was not the deformation value of the rock mass at the last moment. In each rock mass deformation and failure stage, any point in the rock mass was not displaced in a constant direction (rising or sinking). Therefore, the absolute value of the incremental change in displacement is used to characterize the surface displacement of the rock mass during each loading stage.

The rock mass of the 3# test point is the most comprehensive. In this research, the typical dynamic data of the 3# surface deformation was for analyzing the progressive failure law of the moderately weathered red mudstone foundation.

**Figure 12:**
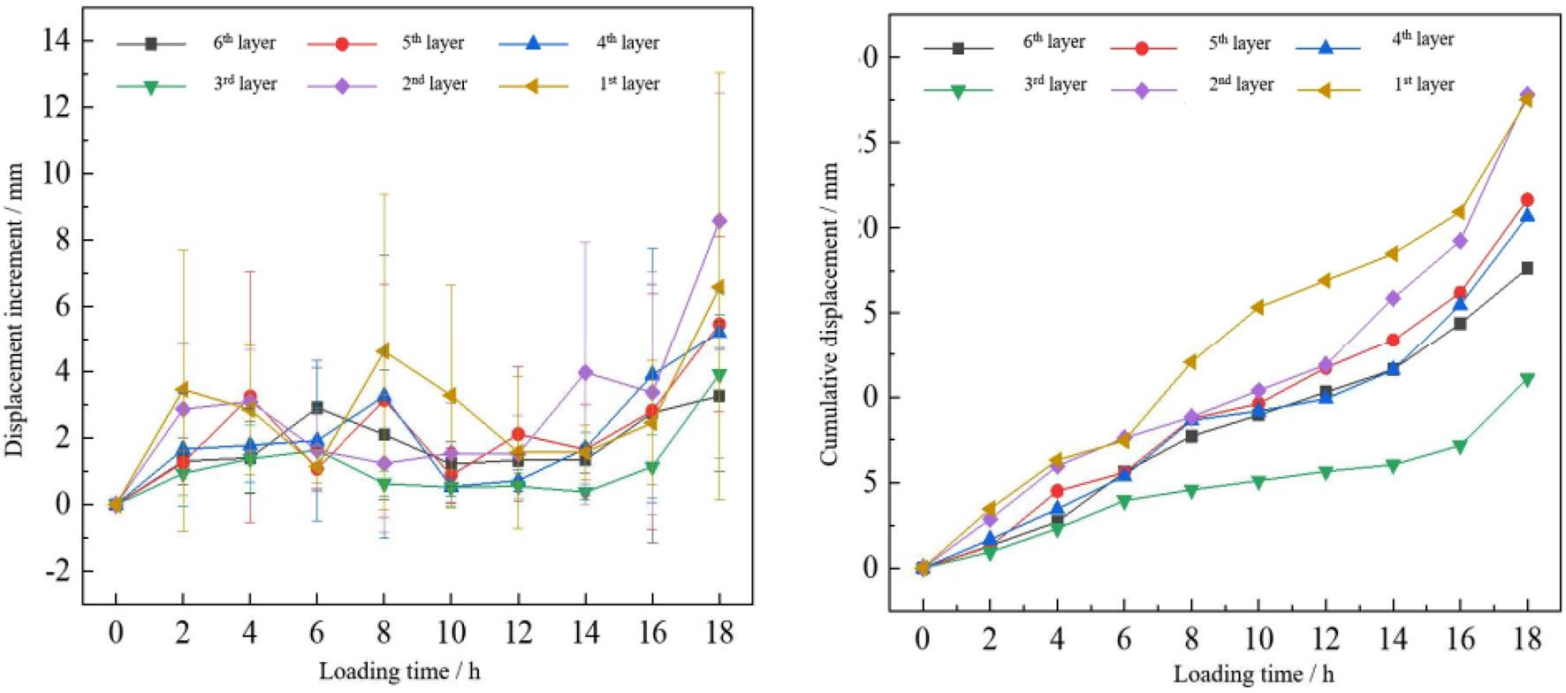
Spatial displacement of the 3# test point.

Figure 12 shows the displacement increment and accumulated deformation of 3 # test points. The displacement increment of each layer presents a "fluctuation” phenomenon transmitted from near to far over time.

The first layer was used as an example in this research. As the top loading is increased from 2000 to 3200 kPa, the strain increases and reaches the first peak value of 3.20 mm. Then, as the top load reached 3200–4600 kPa, the strain increments gradually decreased to 1.01 mm. When the top load reached 5000 kPa, the strain increment reached the second peak and decayed gradually up to 5800 kPa. Finally, when the load was increased to 6000 kPa, the strain increments suddenly increased to 5.84 mm, at which point the rock mass failed. Spatial ripples peaked between 1.63 and 4.61 mm. In addition, wave valleys varied between 0.38 and 1.41 mm. Comparison of the displacement increments of different rock masses at each time showed that the displacement increments of the layers near the bearing plate were generally greater than those of the other layers. The third circle as the smallest displacement increment. At the top load of 6000 kPa, the displacement increments of all layers started to exhibit a rapid growth trend overall. For spatial directions, the range of the peak strain increment was between 3.25 and 8.58 mm. The cumulative displacement corresponded to the increment in displacement, and the situation was similar to that above. It continued to grow at different slopes and reached maximum value. The peak cumulative displacement values in space were 11.10 to 27.83 mm. The whole failure process is in accordance with the stress deformation and failure process of rock mass under different load conditions, namely compaction stage, elastic deformation stage, stable fracture development stage, unstable fracture development stage, loss of strength and complete failure stage. The loads corresponding to each stage are 2–4 MPa, 4–5 MPa, 5–5.8 MPa, 5.8–6.2 MPa, 6.2–7.4 MPa, respectively.

## Discussion

### Failure analysis

**Figure 13:**
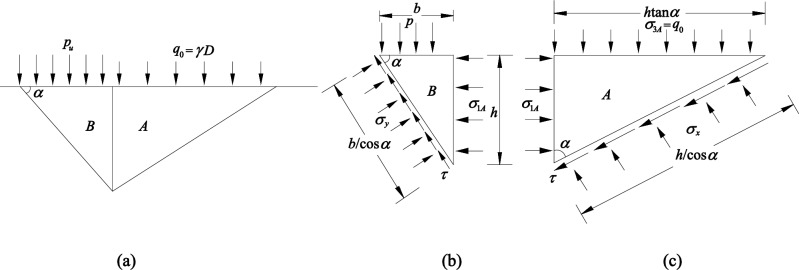
Shear failure model of rock mass. (**a**) Failure model, (**b**) active Rankin wedge, (**c**) passive Rankin wedge^9^.

This paper proves the rock mass failure model by contradiction. Assuming that all test sites are shear failure model as shown in Fig. 13, according to the Hoek–Brown Failure Criterion^9,43,44^, this simplified model reduces the rock-base plastic region to active Rankine zone and passive Rankine zone^9^. The assumption is made that there is no shear stress on the boundary between the active and passive wedges as well as on the bearing surface. In this study, passive wedges were not affected by supplementary pressure, and the footing base pressure of the active wedge is p. The theoretical limit of internal friction angle of the rock mass (*φ*_2_) can be obtained by failure zone diameter D_1_ and failure depth H of rock mass based on Eq. (6). The theoretical range of rock mass failure D_2_ can be calculated using the friction angle (*φ*_*1*_) by triaxial compression test and failure depth H based on Eq. (7).

6 $$ \varphi_{2} = 2\left( {{\text{arctan}}\frac{{D_{1} }}{H} - 45^{ \circ } } \right) $$

7 $$ D_{2} = H\tan \left( {\frac{{\varphi_{1} }}{2} + 45^{ \circ } } \right) $$

In accordance with the formula above, the results of the theoretical calculation are compared with the experimental condition, and theoretical and practical differences are shown at all points in Table 3, which may rule out the shear failure mode.

**Table 3:** The differences of theoretical and practical results.

No.	Theoretical results				Practical results					Differences (%)
	*φ*_*1*_/°	*φ2*/°	H/cm	D_2_/cm	D_1_/cm (The starting point is the outer boundary of the bearing plate)					
					Measurement range				Average of D_1_	
					1	2	3	4		
1#	31.3	37.8	10.0	17.8	19.7	15.2	20.1	26.7	20.4	12.7
2#		77.2	4.0	7.1	31.9	34.9	48.5	27.6	35.7	80.1
3#		66.1	7.0	12.5	29.4	32.1	27.4	43.3	33.1	62.3

**Figure 14:**
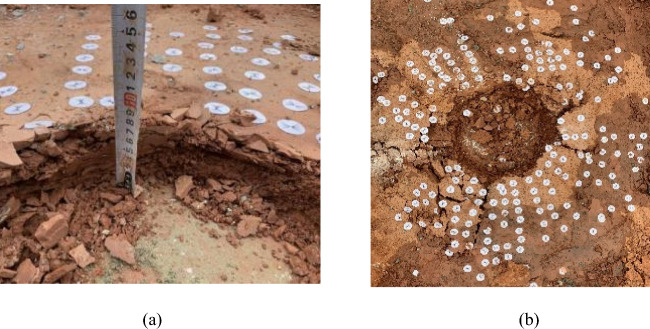
Rock failure details. (**a**) spike marks and (**b**) surface cracks

A detailed summary of the rock mass failure characteristics at the test site in the research area is presented. (1) The rock mass at the test site is a thin rock mass of 7–10 cm thickness. Under vertical load, the vertical puncture mark of rock near the outer edge of the bearing plate is obvious, as seen in Fig. 14a. (2) The rock mass around the plate deforms and ruptures more uniformly, primarily is the surface symmetric tensile crack, and the depth of crack extension is consistent with the thickness of the rock layer, as seen in Fig. 14b. (3) No significantly uplift or collapse settlement is evident in the rock mass surrounding the plate, and there has been no continuous sliding failure of the plate edge.

**Figure 15:**
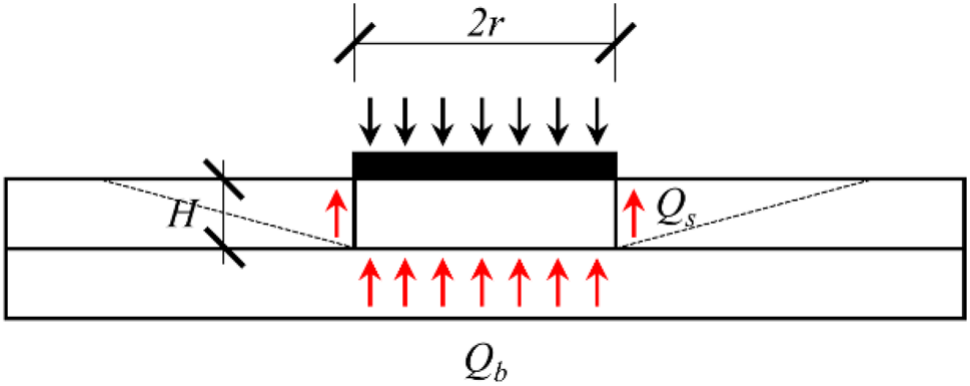
The punching failure mode.

In summary, failure locus of moderate weathered mudstone with layered structure can be attributed to the increasing load under the load plate, which causes a gradual increase in rock mass deformation. If the load exceeds a certain value, the mass of rock around the plate will be subjected to vertical shear and damage will take place. Under load, the rock mass does not form a destructive wedge, and the angle between the horizontal bearing surface and the active wedge does not satisfy shear failure model of the isotropic rock mass with the largest difference close to 80% (As seen in Table 3). At the same time, the deformation of the rock mass outside the plate is more uniform, and the failure mode of the point rock foundation is regarded as a kind of punching failure mode, the process of which can be seen in Fig. 15.

In punching failure mode, the load is only able to damage a layer of rock mass in contact with the bearing plate, its effect is minimal on the mass of rocks that are not in direct contact. Therefore, the main factor limiting the bearing capacity of rock mass is its compressive strength. Three assumptions can be made in exploring the computational method:

1. The bearing plate will not deform during load testing, so it can be regarded as a rigid thin layer;
2. The rock mass within the bearing plate action is compressed downward and deformed outward during the loading process, whereas the rock mass at the bearing plate edge is sheared away;
3. As the load is increased, the mass of rock in the cylindrical region of the bearing plate breaks down and is eventually crushed;
4. The load-bearing process is compared to the frication & end-bearing piles, i. e. the top load is jointly supported by the lateral friction of the cylinder (*Q*_*s*_) and the rock mass compressive strength (*Q*_*b*_).

In this way, the force balance equation of weathered mudstone in a thrust failure mode is established. Referring to Eq. (8).

8 $$ Q_{u} = Q_{s} + Q_{b} = \psi_{s} (2\pi rH\tau + \pi r^{2} \sigma_{c} ) $$

where *r* is the diameter of the bearing plate; *H* is the depth of failure; *τ* is the shear strength under confining pressure; *σ*_*c*_ is the peak strength under triaxial compression; *ψ*_*s*_ and *ψ*_*b*_ are the correction factor of shear strength and compressive strength.

During the loading process, the rock mass in the lower portion of the bearing plate is deformed outward, causing an increase in the normal stress of the shear portion. Thus, the present-depth horizontal geo-stress can simply be used as the normal stress in the calculation. Likewise, *σ*_*c*_ can also be obtained from a uniaxial compressive strength. Equation (8) is simplified to Eq. (9) for BPT of rock:

9 $$ f_{ak} { = }\frac{{1}}{{3}}\psi_{s}^{\prime } \left( {\frac{2H}{r}\tau + f_{a} } \right) $$

where *f*_ak_ is characteristic value of subgrade bearing capacity; *H* is the depth of failure; *τ* is the shear strength of the rock, it can be calculated by Eq. (10); *f*_*a*_ is uniaxial compressive strength of rock; *ψ*_s_, is the rock mass resistance correction coefficient in the cylindrical area of the bearing plate, $\psi_{s}^{\prime } = \psi_{s} \cdot \pi r^{2}$; 1/3 is Load Capacity Safety Factor for rock foundations, which is determined based on guideline DB51/T5026^2^ and GB50007^3^.

10 $$ \sigma_{x} = \sigma_{y} = \frac{\mu \gamma h}{{1 - \mu }} $$

where μ is Poisson’s ratio; γ is natural gravity; h is testing depth.

To replace the load carried by the shear part, the calculation formula can be multiplying the uniaxial compressive strength of rock by the correction factor, which can reduce the difficulty of calculating the parameters. Equation (9) is simplified to Eq. (11).

11 $$ f_{ak} { = }\frac{{1}}{{3}}\psi_{s}^{\prime } \left( {\frac{2H}{r}\tau + f_{a} } \right) = \psi f_{a} $$

**Figure 16:**
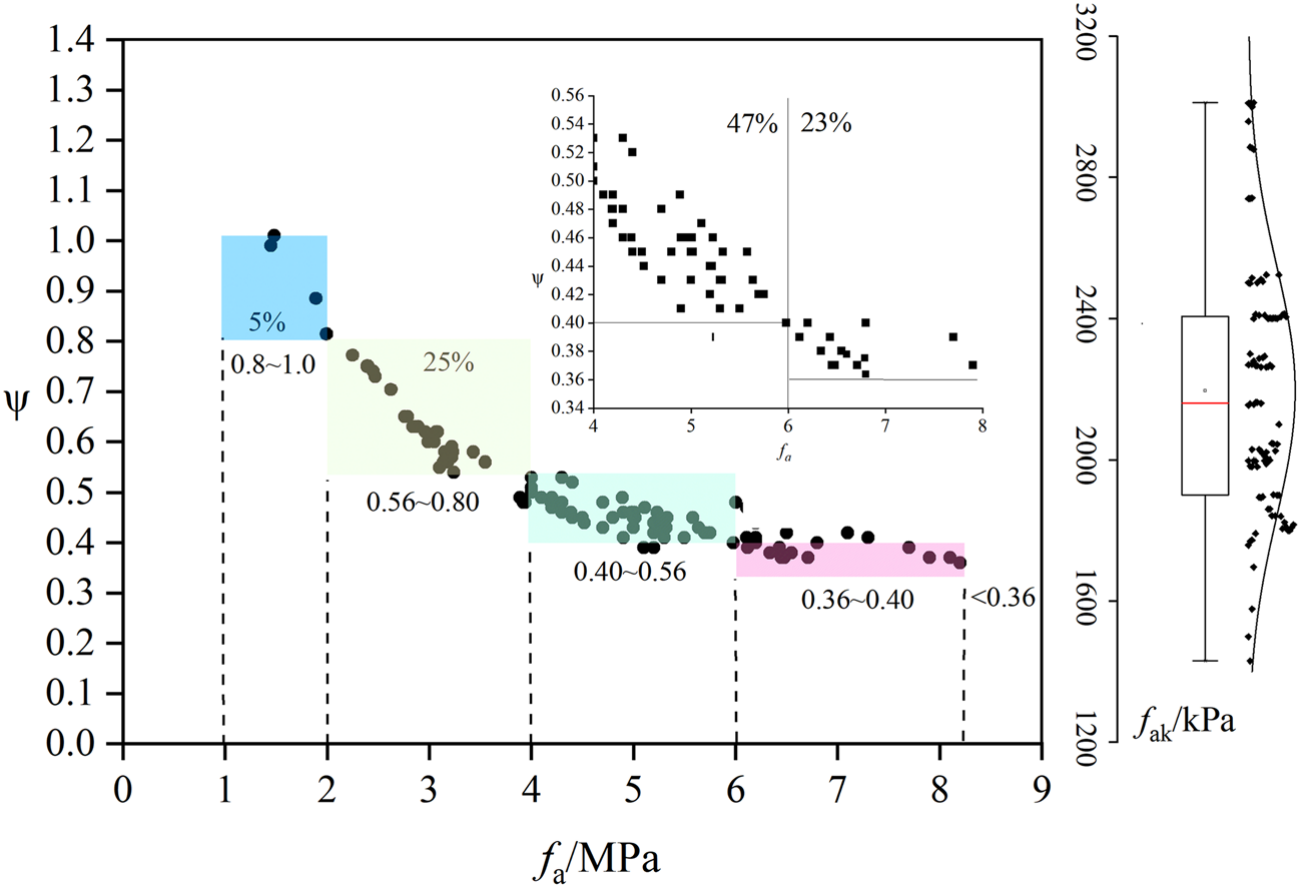
The relationship between *f*_a_ and *ψ.*

This study reveals the potential implications of the reduction coefficient of uniaxial compressive strength, including the influence of rock thickness, rock integrity, the edge shear effect of the pressure plate, and the encircling enhancement effect. In the computational method of this paper, it is very important to obtain the value of parameter *ψ.* In this paper, a detailed analysis of ψ value of moderate weathered mudstone in Chengdu is presented in which the results of 100 on-site load tests of the moderate weathered mudstone foundations and the uniaxial compressive strength tests were compared in 25 engineering cases. The results of the comparative analysis of the two methods are shown in Table 4 and Fig. 16.

**Table 4:** Results of in-situ load tests and uniaxial compressive strength.

Longitude	Latitude	*f*_*a*_	*f*_*ak*_	*ψ*	Displacement/mm	Test number
104.04581	30.74289	4	2044	0.51	5.42	3
104.10194	30.65984	4	1981	0.49	6.76	3
104.02361	30.88173	3.05	1841	0.60	3.99	8
103.91241	30.61023	5	2263	0.45	18.37	8
104.05897	30.60428	5	2157	0.43	3.64	4
104.08057	30.65164	3.07	1900	0.62	3.52	3
104.06684	30.65196	3.43	1990	0.58	3.22	2
104.06679	30.65307	6	2880	0.48	5.14	3
104.07223	30.55181	1.48	843	0.57	10.41	3
104.07133	30.66384	7.3	3010	0.41	0.562	3
104.1318	30.60495	7.9	2959	0.37	8.3	4
104.10194	30.65984	6.8	2740	0.40	10.7	3
104.07964	30.59402	4.5	2021	0.45	6.6	7
104.07993	30.55653	2.79	1800	0.65	33.19	8
103.91404	30.61089	3.92	1894	0.48	5.29	3
104.19363	30.56694	5.98	2400	0.40	5.5	3
104.07054	30.43763	6.48	2400	0.37	7.32	3
104.00487	30.72595	4.99	2290	0.46	1.42	4
104.10194	30.65984	5.64	2400	0.43	5.98	6
104.27763	30.55968	5.58	2524	0.45	17.5	1
104.10367	30.65539	4.39	2000	0.50	1.16	3
104.06226	30.5688	6.55	2500	0.38	4.26	6
104.04717	30.62735	5.74	2400	0.42	2.2	3
104.27091	30.72079	3.1	1697	0.55	3.72	6

*f*_*a*_: Standard values of rock uniaxial compressive strength(MPa); *f*_*ak*_: Characteristics values of rock uniaxial compressive strength(kPa); *ψ*:strength reduction factor.

The data of *f*_ak_ in the table is the mean of the tests.

There are 100 groups in the dataset for this collection experiment, with a typical range of load-bearing values of 1400–300 kPa for the mudstone bedrock with a 1700–2400 kPa of 75%. The box plot can be viewed in Fig. 16. As can be seen from the Median and Inter Quartile Range in the Box-plot, there is a high degree of symmetry in the data distribution. The data collected tend to be well normally distribution, and there is no effect of outliers. Indicating that no mild or extreme outliers exist in the analysis dataset.

Defines strength reduction factor as the ratio of *f*_ak_/*f*_a_. As can be seen in Fig. 16, the macroscopic trend of the relationship approximates the hyperbolic variation and the coefficient of reduction decreases with increasing uniaxial compressive strength. The coefficient of reduction varies between 0.36 and 0.80, with an overall value that is larger than the maximum recommended by the specification^2^ of 0.5. Based on the curve’s trend, the relationship can be split into 4 segments, which are 1–2 MPa, 2–4 MPa, 4–6 MPa, 6–8 MPa, respectively. The strength reduction factor for each interval is derived from Eq. (12).

12 $$ \psi  = \left\{ {\begin{array}{*{20}l}    {1,f_{a}  \le 1;} \hfill  \\    {[0.8 - 1),\;1 < f_{a}  \le 2;} \hfill  \\    {[0.56,0.8),\;2 < f_{a}  \le 4;} \hfill  \\    {[0.56,0.4),\;4 < f_{a}  \le 6;} \hfill  \\    {[0.36,0.4),\;6 < f_{a}  \le 8;} \hfill  \\    { < 0.36,\;f_{a}  > 8} \hfill  \\   \end{array} } \right. $$

These four intervals contain 5%, 25%, 47%, and 23% of data, respectively. It should be noted that the statistical results presented are relatively small for rock mass test specimens with uniaxial compressive strength less than 1 MPa and greater than 8 MPa, because of limitations in the uniaxial compressive strength of the medium-weathering mudstone that are rare in cases less than 1 MPa and greater than 8 MPa. The high complexity and realism of the data structures is similar to the in-situ experimental data distribution structures. The strength reduction factor is thus 0.36 ∈ (*f*_a_ > 8) and 1 ∈ (*f*_a_ < 1), respectively, for contingencies beyond the statistical range. Furthermore, although the relation between *f*_a_ and *ψ* decreases macroscopically in hyperbolic form. But the tendency for two relations at each interval to decrease nearly linearly. Therefore, the parameters within the interval are proposed to be determined by linear interpolation method. It is important to note that the moderate weathered mudstone fractures in Chengdu are relatively developed and susceptible to weathering. On the other hand, most of the above statistical results are obtained under experimental conditions with no loading in the rock foundation and still have an impact on the effective reduction coefficient. Future studies will continue to attempt to collect more experimental data and provide more specific recommendations.

### Application and validation

**Figure 17:**
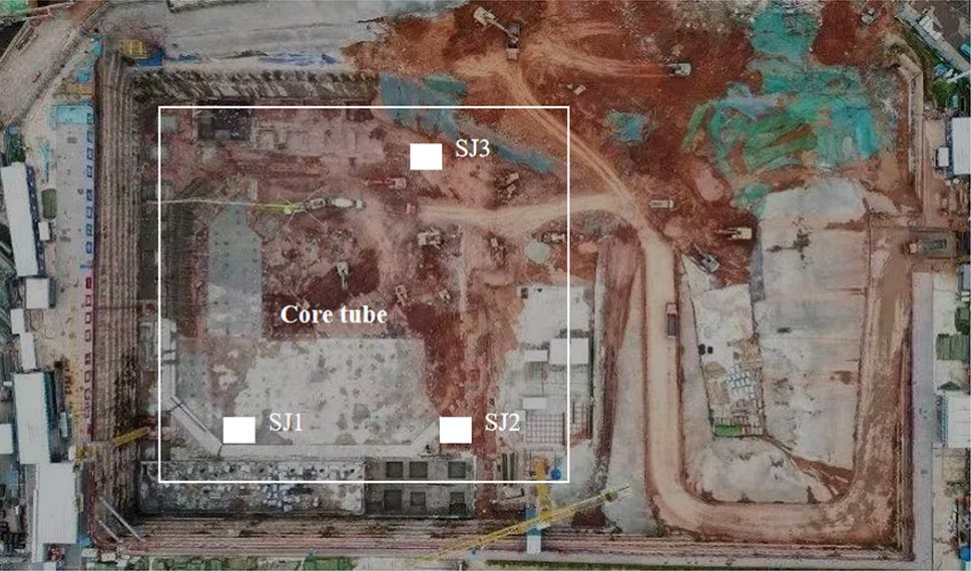
Schematic diagram of test sites.

An example is given of a super-high rise building in Chengdu region. The project is located in the central business district of Qinhuangsi Temple in Tianfu New District, south of Chengdu. Medium weathering mudstone with a natural density of 2.48 g/cm^3^ is used as the base retaining layer of the building, with an average uniaxial compressive strength of 6.00 MPa in the native condition. For the triaxial natural shear strength parameters, the mean internal angle of friction and cohesion were 37.9° and 0.8 MPa, whereas rock mass wave speeds were measured from 2200 to 3200 m/s on the basis of borehole wave speeds. Three deep well (numbered SJ01, SJ02, SJ03) excavated. Figure 17 shows the test site.

**Figure 18:**
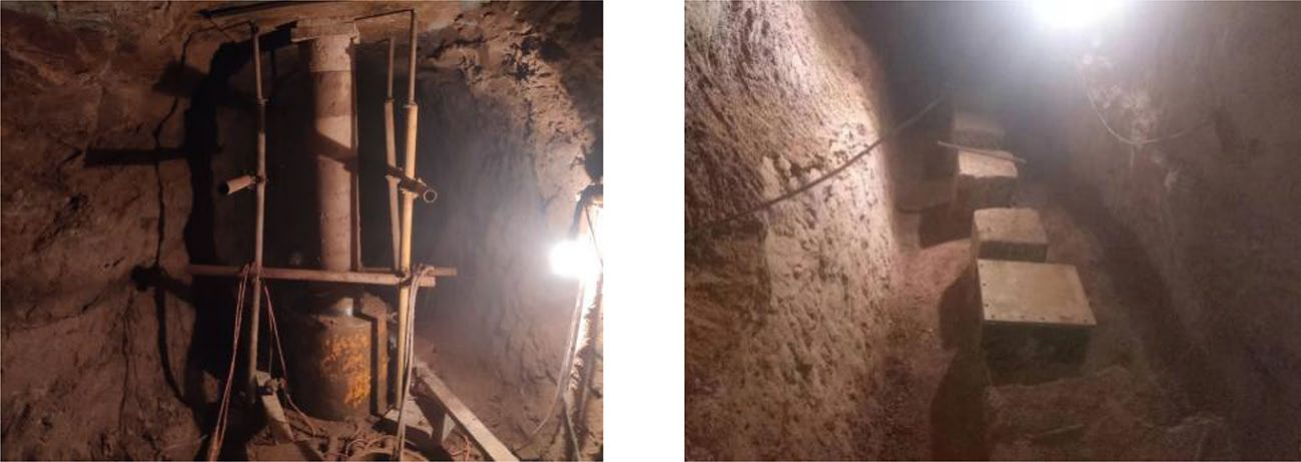
Photos of bearing plate tests.

In Fig. 17, the depth of SJ01 is 30 m (459.06 m high), that of SJ02 is 36 m (451.15 m high), SJ03 has a depth of 39 m (447.57 m high), and the bottom of the well is excavated in an adit measuring 2 m wide by 2 m high by 8 m deep. Load tests with a bearing plate diameter of R = 300 mm, 500 mm, and 800 mm were carried out in each adit, totally in 9 groups. The operation of the load test strict reference specification^3^, after digging to the target layer, the test point is coarsely leveled. Then the pressure plate, jack, binocular vision measurement system, and other test equipment are placed sequentially. Loading can be applied by the slow maintenance loading method, which is divided into 8 to 12 stages. Photos of bearing plate tests can be seen in Fig. 18.

The results of the experiment are shown in Table 5.

**Table 5:** The in-situ test values of bearing capacity of moderately weathered mudstone in the study area.

Test no.		In-situ test			*Kv*
Well no.	Plate diameter /mm	P_a_/kPa	*f*_ak_/kPa	Final load/kPa	
SJ01 (459.06 m)	300	3000	2400	9000	0.46
	500	2400	2400	9600	0.46
	800	2700	2300	6300	0.43
SJ02 (451.15 m)	300	2400	2100	7800	0.39
	500	2400	2000	10,800	0.39
	800	2700	2100	11,700	0.39
SJ03 (447.54 m)	300	3600	2800	11,800	0.49
	500	3000	3000	9600	0.50
	800	3600	2700	10,800	0.43

From the Table 5, for the same level adit, the difference in the bearing characteristics of the foundation is less than 10%. In particular, the mudstone bearing capacity in the deep well SJ01 is 2300–2400 kPa, in SJ02, the depth of the well is 2000–2100 kPa, in SJ03, it is 2700–3000 kPa. This phenomenon shows that the size of the load plate has little effect on the load carrying capacity of mudstone foundations that have similar characteristics. The different adit experiments give different results, which is related to the individual difference in the mass of the rock, especially the integrity of the rock mass and the mineral composition. In the case of SJ01, SJ02, SJ03 adit, the mudstone rock mass integrity index (*K*_v_) was found to be 0.43–0.46, 0.39, 0.43–0.50, respectively. The mineral content of the clay was found to be 29–30%, 36–47%, 17–25% respectively. In addition, the difference in the load carrying capacity of the rock foundation is caused by the combination of environmental conditions and human error in the operation.

Specimens were taken at the same time near the plate test point in three deep wells to test the uniaxial compressive strength, and the modified method of reduction coefficients was validated. The comparative calculation was performed using Eq. (12), and the experimental and validation results are shown in Table 6. Based on the computational results, it can be seen that the load carrying capacity of the soft rock foundation estimated by the coefficient of reduction method proposed in this paper is similar to that of the BPT. With less than a 5% difference between the 2 methods and good effect sizes, which can be used as the basis for the carrying capacity calculation. Depending on the partitioning of the uniaxial compressive strength test, the correction coefficient may be selected according to Fig. 15, and the range of values may be determined by linear interpolation.

**Table 6:** The comparative calculation by recommended methodology.

Site	No.	*f*_*a*_*/*kPa		*f*_*rk*_ /MPa	*f*_*rk*_ of average /MPa	*ψ*	Calculated *f*_ak_/kPa	Difference (%)
		Single-point results	Average value					
Case site	1	1595	1640	2.73	2.73	0.6	1638	0.5
	2	1567		2.67				
	3	1758		2.79				
SJ01	1	2400	2367	4.9	5.96	0.4	2384	0.7
	2	2400		5.8				
	3	2300		7.19				
SJ02	1	2100	2103	3.47	4	0.5	2000	4.9
	2	2000		3.78				
	3	2100		4.39				
SJ03	1	2800	2800	6.89	7.1	0.39	2769	1.1
	2	3000		8.31				
	3	2700		8.41				

## Conclusion

In order to more scientifically determine the load carrying capacity of the medium weathered mudstone foundation in Chengdu area, in this paper, a case study of the ground foundation project of a supertall building in Tianfu New District of Chengdu was presented. Based on the BPT with BVM method, the process of deforming the mudstone foundation under a vertical load is dynamically tracked, and an empirical formula for calculating the load bearing capacity of this type of bedrock foundation is proposed.

1. Based on failure state and displacement increments, the progressive failure of moderately weathered red mudstone foundation is characterized. The load increases, the increment displacement presents a wave format at each moment, and there is a wave peak transfer phenomenon into the distance. At the moment of failure, the increment displacement increases sharply and the surface deformation accelerates.
2. The failure mode of vertical load-bearing foundations can be reduced to that of punching failures for thin moderate weathered mudstone bedded structure. A key feature of this process is the rock mass within the bearing plate action is compressed downward and deformed outward during the loading process, whereas the rock mass at the bearing plate edge is sheared away.
3. The force balance equation of weathered red mudstone in a punching failure mode is established, it reveals the potential implications of the reduction coefficient of uniaxial compressive strength. Then based on the results of 100 in situ load tests and the uniaxial compressive strength tests were compared in 25 cases of engineering. By comparison, a proposal for the value of key computational parameters is proposed. The formula presented in this paper is proven to be suitable for engineering applications.

### Recommendations

Overall, the research focus of this paper is on the load bearing capacity of medium weathered mudstone bedrock of layered isotropic. Based on the analysis of 100 in situ loading tests and the uniaxial compressive strength test case under the same condition, the result is a revised method of calculating the bearing capacity of the foundation. It should be noted, however, that the parameters used in the computation are the uniaxial compressive strength of the rock in its native state. Moderately weathered mudstones are susceptible to weathering in practical engineering, particularly water immersion, which requires the reduction factor to be properly accounted for, and the magnitude of this reduction needs to be explored. In addition, due to the lack of test specimens with other layered isotropic rock mass, the range of reduction coefficients was provisionally between 0.36 and 0.8. Further research is needed to gather more experimental data to explore how different environmental factors affect the accuracy of the bearing capacity of the rock and to further revise the method of calculation.

## Data Availability

All data, models, and code generated or used during the study appear in the submitted article.
